# Better Together: Current Insights Into Phagosome-Lysosome Fusion

**DOI:** 10.3389/fimmu.2021.636078

**Published:** 2021-02-25

**Authors:** Jenny A. Nguyen, Robin M. Yates

**Affiliations:** ^1^ Department of Biochemistry and Molecular Biology, Cumming School of Medicine, University of Calgary, Calgary, AB, Canada; ^2^ Department of Comparative Biology and Experimental Medicine, Faculty of Veterinary Medicine, University of Calgary, Calgary, AB, Canada; ^3^ Cumming School of Medicine, Snyder Institute of Chronic Disease, University of Calgary, Calgary, AB, Canada

**Keywords:** phagosome-lysosome fusion, phagosome, phagocyte, lysosome, membrane fusion, microbial clearance, homeostasis, phagosome maturation

## Abstract

Following phagocytosis, the nascent phagosome undergoes maturation to become a phagolysosome with an acidic, hydrolytic, and often oxidative lumen that can efficiently kill and digest engulfed microbes, cells, and debris. The fusion of phagosomes with lysosomes is a principal driver of phagosomal maturation and is targeted by several adapted intracellular pathogens. Impairment of this process has significant consequences for microbial infection, tissue inflammation, the onset of adaptive immunity, and disease. Given the importance of phagosome-lysosome fusion to phagocyte function and the many virulence factors that target it, it is unsurprising that multiple molecular pathways have evolved to mediate this essential process. While the full range of these pathways has yet to be fully characterized, several pathways involving proteins such as members of the Rab GTPases, tethering factors and SNAREs have been identified. Here, we summarize the current state of knowledge to clarify the ambiguities in the field and construct a more comprehensive phagolysosome formation model. Lastly, we discuss how other cellular pathways help support phagolysosome biogenesis and, consequently, phagocyte function.

## Introduction

Professional phagocytes, such as macrophages, dendritic cells (DCs), and neutrophils, are critical to the innate immune response and the maintenance of homeostasis through their ability to ingest and degrade microbes, debris, and dying cells (1). The versatility of professional phagocytes stems from the expression of phagocytic receptors that can mediate the uptake of a vast array of cargoes *via* phagocytosis. Following uptake, the engulfed material is contained within the specialized vacuole called the phagosome, which initially has the lumenal characteristics of the extracellular space (2). The phagosome undergoes progressive maturation through multiple transient fusion-fission events with vesicles of the endolyososmal system leading to an increasingly acidic and hydrolytic environment within the phagosomal lumen (3, 4). The fusion of the phagosome with lysosomes forms the mature phagolysosome (PL) which has full degradative and microbicidal capacity. Heterotypic fusion between the phagosome and lysosome is imperative for phagocytes to carry out their functions in immunity and homeostasis and is a tightly regulated process. The importance of membrane fusion in phagocyte function is further highlighted by the myriad of mechanisms various pathogens have developed which impede fusion machinery in order to prevent PL formation, and the microbicidal environment that is established. Indeed, gram-negative bacteria *Mycobacterium tuberculosis* and *Coxiella burnetii*, parasites of the *Leishmania* genus and the fungi *Aspergillus fumigatus* can inhibit PL fusion to avoid death in phagosomes, among many others (5–9).

PL fusion involves the stepwise recruitment and coordinated action of a number of proteins. To date, 30 soluble N-ethylmaleimide-sensitive fusion factor attachment protein (SNAP)-receptors (SNAREs), 20 Rab GTPases, six multi-subunit tethering complexes, and four tethering proteins of the Sec1/Munc18 (SM) family have been identified on phagosomes (10, 11). The extensive number of fusion proteins and promiscuity among interacting components creates mechanistic redundancy of this crucial function of phagocytes. Given the overwhelming number of fusion proteins and the even larger pool of potential binding partners, it is unsurprising that the identity and roles of the fusion machinery, their regulatory signals, and their spatiotemporal organizations in PL fusion have yet to be fully elucidated. These gaps in the literature continue to be active areas of investigation, and here we highlight the progress made on this topic in the context of phagocyte biology. This review aims to summarize current and recent developments in understanding the fusion machinery modulating PL fusion and the consequences to phagocyte function when this machinery is impaired. Finally, we discuss how phagocytes can employ the related engulfment process of autophagy, to support the phagocytic pathway during homeostasis and defense against pathogens.

## Phagosome-Lysosome Membrane Fusion: An Overview

Once the phagosome is formed and has undergone transient fusion-fission events with early and late endosomes, phagosomes migrate to the lysosome-rich perinuclear region of the phagocytic cell (12). Similar to many other types of cells, phagocytes employ a coordinated transport system consisting of the microtubule (MT) networks, the associated MT motors, and their effector/adaptor proteins to carry phagosomal vesicles to the perinuclear region (13, 14). Long-distance transport is initiated by the small Rab GTPases Rab5 and Rab7 which are recruited to early and late phagosomes, respectively. Rab5 and Rab7, in turn, coordinate the recruitment of necessary motor proteins required for the dynein-driven transport of phagosomes to the perinuclear region (13, 15). Once phagosomes and lysosomes are in close apposition, membrane fusion occurs through the concerted action of fusion proteins.

**Figure 1:**
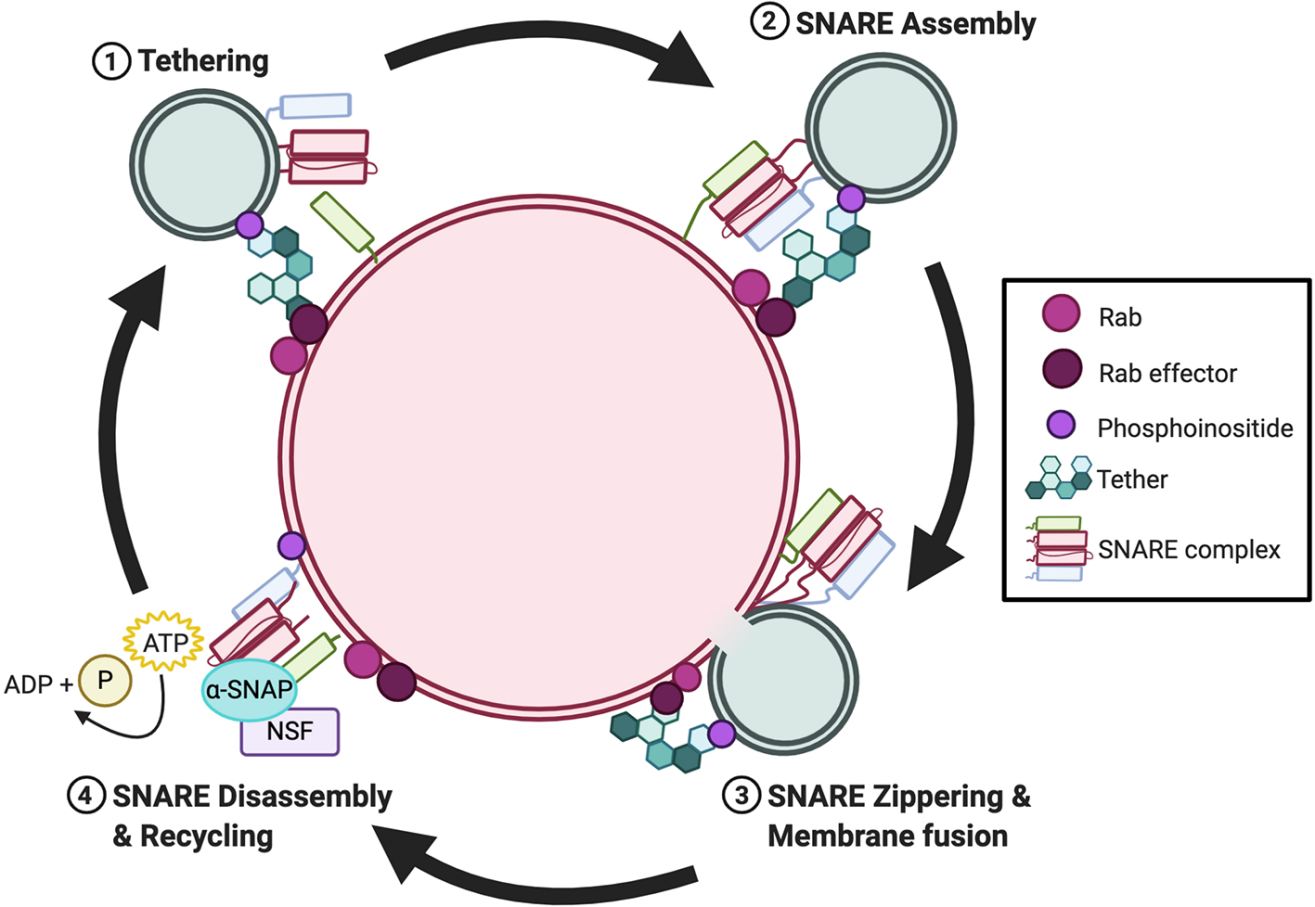
Overview of the cyclical nature of membrane fusion. Rab GTPases and their effectors recruit tethers to the membrane fusion site during the tethering step. Subsequently, SNAREs are recruited to opposing membranes and assemble in *trans via* the catalyzing activity of a SM protein. Once assembled, SNARE zippering drives membrane fusion. Post-fusion, NSF and α-SNAP bind to the *cis*-SNARE and ATP-driven complex disassembly recycles the individual SNAREs intothe cytosol for recycling.

Membrane fusion is the process by which two separate lipid membranes combine to form one continuous bilayer (16). Within the endomembrane system, fusion requires the coordinated action between members of the Rab GTPase superfamily and their effectors, tethering factors, N-ethylmaleimide-sensitive factor (NSF), and SNAREs (17). Generally, these proteins are consecutively recruited during the following steps of membrane fusion: i) tethering; ii) SNARE assembly; iii) SNARE zippering and membrane fusion; and iv) SNARE disassembly and recycling (**Figure 1**). First, Rab GTPases are recruited to the membrane of vesicles pending fusion, to mark the position for fusion. Subsequently, Rabs, through the recruitment of their effectors bind to tethering factors from the cytosol (18, 19). Through this action, Rabs establish the site at which proteins assemble into membrane microdomains at the fusion site. Rab effector-mediated tethering brings adjacent membranes into proximity. Once tethered, SNARE proteins congregate at the fusion site and supply the energy necessary to overcome the electrostatic repulsion between two lipid membranes (20–22). Numerous SNAREs reside on endomembranes, yet only a finite number can form a stable SNARE complex in *trans* that consists of four SNARE domains (23). SNARE assembly is not a spontaneous process but requires SM proteins to form SNARE intermediates that await a missing cognate SNARE (24, 25). Once a *trans*-SNARE complex is formed, a conformational change brings the two membranes together, forming a *cis*-SNARE complex in a process called “zippering” (20–22, 26). The post-fusion *cis*-SNARE complex, where all SNARE proteins are located on the same membrane, is then disassembled by NSF along with its cofactor α-SNAP (27–29). The disassembly process is powered by NSF-mediated ATP hydrolysis which provides the energy for the dissociated SNARE proteins.

Early studies on pathogen clearance and efferocytosis in mice and *Caenorhabditis elegans* identified specific Rab GTPases and their corresponding tethering complexes in PL biogenesis and phagocyte function (30–33). Despite their lack in professional phagocytes, *C. elegans* have phagocytic-like cells that have been used in many early and current studies to study phagosome maturation after efferocytosis due to their genetic conservation (34–36). Thus, *C. elegans* has been a powerful model to identify genes and mechanisms involved in PL fusion.

Identifying SNAREs involved in PL fusion has been more challenging due to their promiscuous nature and involvement in several cellular pathways crucial for cellular upkeep. Although, putative SNARE complexes have recently been identified using cell-free systems where subsequent investigations are beginning to unravel the functions of these proteins in PL fusion (37–40). Other insights into the proteins modulating PL fusion have been inferred from studies of the closely related process of endo-lysosome fusion in other cells. However, differences in the cargo and regulation between phagocytosis and endocytosis, maintain PL fusion as a distinct process compared to endosome-lysosome fusion (41). Given the large degree of heterogeneity in models and approaches to study this process, this review aims to consolidate the literature focusing on PL fusion proteins in phagocyte-specific studies, and where information is lacking in this context, identify potential candidate proteins and complexes, in order to generate a clearer picture of this fundamentally important aspect of phagocyte function. The key components of the PL fusion machinery are summarized in **Supplementary Table 1**.

## Marking the Membranes: Small Rab GTPases

The Rab GTPases are peripheral membrane proteins that serve as molecular identifiers on membranes, and to regulate multiple steps during membrane fusion. Rab GTPases function as molecular switches that toggle between an inactive GDP-bound and an active GTP-bound state. Rabs are maintained in their inactive soluble GDP-bound state by a guanine dissociation inhibitor (GDI) (42, 43). At vesicular membranes, guanine exchange factors (GEFs) possess the dual capacity to recruit Rabs from the cytosol and catalyze the displacement of GDP with GTP, which both activates Rabs and displaces the GDI, thus allowing for Rabs to be inserted into the membrane (44, 45). Once activated, Rabs are able to bind to effector proteins on fusion targets in order to tether fusing vesicles, modulate the recruitment of fusion components, and mediate the process of PL biogenesis. Post-fusion, GTPase-activating proteins (GAPs) hydrolyze GTP to inactivate Rabs where they are solubilized by a GDI, returning them to the pool of inactive cytosolic Rabs (46). The numerous influences on GTPase regulation make Rabs a strong focal point for modulation of PL biogenesis.

It has long been established that small GTPase Rab7 is essential for the fusion between late-stage phagosomes and lysosomes, and consequently, for the function of phagocytes in both pathogen and apoptotic cell clearance (33, 47, 48). Several pathogens have been described to inhibit Rab7 recruitment in order to replicate and survive within phagosomes, and has been comprehensively reviewed (49). Early studies reported the inhibition of Rab7 recruitment to the phagosomes by *M. tuberculosis* and *M. bovis* J774 macrophages, preventing PL fusion (33). A more recent example describes the necessity of Rab7 in the clearance of *Pseudomonas aeruginosa* infection in J774A.1-derived macrophages (50). This study reported that sialylated *P. aeruginosa* remained viable in phagosomes by preventing the recruitment of Rab7. In addition to the clearance of microbes, Rab7 is essential to the degradation of apoptotic cells following phagocytosis. In a *C. elegans* model of apoptotic cell removal, the siRNA-mediated knockdown of Rab7 contributed to the accumulation of apoptotic bodies in phagosomes (34). Similarly, in an *in vivo* model of murine ulcerative colitis, inflammation and disease severity was exacerbated when Rab7 activation and recruitment was impaired in macrophages, reportedly due to an accumulation of apoptotic epithelial cells in the colon (48).

**Figure 2:**
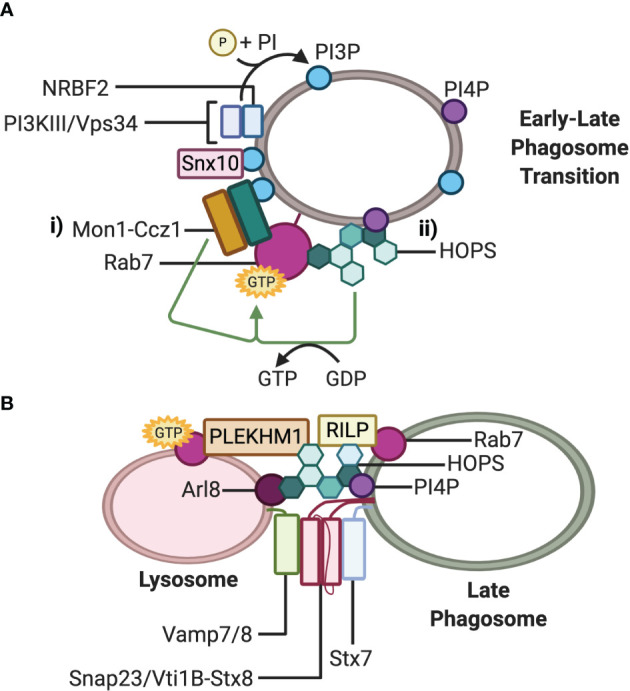
Fusion machinery involved in phagosome maturation. **(A)** Working models for Rab7 recruitment and activation at the phagosome membrane during maturation: i) The Mon1-Ccz1 complex is recruited directly by PI3P and indirectly by Snx10 and NRBF2-PI3KIII/Vps34. PI3P activates the GEF activity of Mon1-Ccz1; ii) The GEF activity of PI4P-recruited HOPS activates Rab7. **(B)** Working model of the *trans*-SNARE complex mediating fusion between the late phagosome and lysosome. The small GTPase Rab7 recruits effectors RILP and Plekhm1, and concurrently with Arl8, additionally recruits the HOPS tethering complex. HOPS docks the late phagosome to the lysosome and stabilizes the *trans*-SNARE Stx7-Snap23-Vamp7/8 or Stx7-Vti1b-Stx8-Vamp7/8 complex at the point of membrane fusion. Note that either Plekhm1, Arl8, PI4P, or RILP on either lysosomes or phagosomes bind to HOPS Vps41 at one time, while RILP can also bind HOPS Vps39.

The mechanism by which Rab7 is recruited to phagosomes in professional phagocytes is not fully elucidated. However, studies in *C. elegans* demonstrated that Rab7 recruitment to late endosomes/lysosomes was facilitated by the cytosolic SAND1-CCZ1 complex (51–53). The mammalian orthologues of SAND1 and CCZ1, namely, Mon1a/b and Ccz1 display GEF activity and were found to form a complex which displaces the GDI associated with the GDP-bound form of Rab7, allowing GTP switching and integration of Rab7 into phagosomal membranes (**Figure 2Ai**) in *C. elegans* which is believed to be evolutionary conserved in mammals (52).

Phosphatidylinositol-3-phosphates (PI3P) on early phagosomes have been proposed to both recruit Mon1-Ccz1 to early-to-late transitional phagosomes and activate the GEF activity of the complex (**Figure 2Ai**) (48, 53). Recently, ﻿nuclear receptor binding factor 2 (NRBF2), a component of the class III phosphoinositide 3-kinase (PI3KIII)/vacuolar sorting protein (Vps) 34 complex that generates PI3P on phagosomes, has been implicated to regulate the function of the Mon1-Ccz1 complex during apoptotic cell clearance in macrophages (48). ﻿NRBF2 is a binding partner of the catalytic subunit of the PI3KIII/Vps34 complex and can facilitate the interaction between PI3KIII/Vps34 and Mon1-Ccz1 (**Figure 2Ai**). Through this interaction, NRBF2 may bring the PI3KIII/Vps34 complex to phagosomes or support the activation of PI3KIII/Vps34, thereby generating PI3P to activate the GEF activity of the Mon1-Ccz1 complex. Further evidence that NRBF2 modulates the activity of the Mon1-Ccz1complex is provided by an observed loss of GTP-bound Rab7 around phagosomes as well as delayed Rab7 recruitment in NRBF2-knockout macrophages (48). Lou and colleagues (54) have proposed that another factor, sorting nexin 10 (Snx10), can promote the recruitment of Mon1-Ccz1 to early endosomes and phagosomes to mediate Rab7 recruitment (**Figure 2Ai**). Snx10 is upregulated in response to TLR signaling and phagocytosis of several different microbes, suggesting that Snx10 has an antimicrobial role (54). Indeed, depletion of Snx10 reduced Rab7 recruitment on bacteria-containing phagosomes. Taken together with the studies in *C. elegans*, Mon1-Ccz1 is likely a major Rab7 GEF on phagosomes in mammalian cells.

While there is ample evidence for the Mon1-Ccz1 complex as a Rab7 GEF on endosomes and phagosomes, it is potentially not the only one. Early insights from studies in yeast and *C. elegans* have implicated that the homotypic fusion and vacuole sorting (HOPS) complex can also act as a Rab7 GEF (**Figure 2Aii**) (52, 55). It was demonstrated by Barry and colleagues (56) that HOPS could function the same way in mammalian cells for Rab7 found on phagosomes in macrophages containing *C. burnetii*. Macrophages phagocytosing an avirulent strain of *C. burnetti* induce p38α-mitogen-activated protein kinase (MAPK)-dependent phosphorylation of the HOPS Vps41 subunit which was demonstrated to be necessary for Rab7 recruitment to endosomal and phagosomal membranes (56). In contrast, the lipopolysaccharide produced by the virulent strain of *C. burnetti* prevented p38α- MAPK activation and phosphorylation of Vps41, allowing bacterial persistence in Rab7-deficient phagosomes. Why Mon1-Ccz1 was unable to act as the Rab7 GEF instead of HOPs in this model is unknown; however, it is possible that Mon1-Ccz1 and the HOPS complex act independently depending on the phagosomal cargo.

Rab7 is vital for PL fusion and therefore phagocytic function, but it is not the only GTPase involved in PL fusion: Rab2 and Rab14 have been shown to possibly function redundantly to each other or in parallel to recruit lysosomes to phagosomes during apoptotic cell degradation in *C. elegans* (31, 32). Rab2 and Rab14 are transiently recruited to phagosomes prior to Rab7, and it is postulated that like Rab7, Rab2 and Rab14 may recruit tethering factors to initiate interaction between phagosomes and lysosomes (31). Interestingly, Rab2 is a direct binding partner of HOPS during autophagosome fusion with lysosomes in *Drosophila melanogaster* cells (57, 58), and thus, the HOPS complex may serve as the effector for both Rab2 and Rab14 to coordinate fusion of lysosomes to phagosomes. Additionally, proteomic studies in an oyster cell model of phagocytosis identified Rab2 and Rab14 in the phagosomal proteome (59). Although Rab2 has not been studied in a mammalian model, the role of Rab14 in PL fusion was partially elucidated in Raw264.7 and J774 murine macrophages infected with the fungal pathogen *Candida albicans*. Knockdown of Rab14 disrupted fusion of lysosomes to phagosomes which both delayed the acquisition and activation of hydrolytic proteases in PLs. This defect in lysosome fusion and protease activation resulted in increased susceptibility of macrophages to *C. albicans* (60).

Taken together, Rab2 and Rab14 may function redundantly in an early tethering step prior to Rab7 recruitment and the docking/fusion of lysosomes with phagosomes. The presence of Rab7 on the phagosome membrane sets the stage for PL fusion; however, the mere presence of Rab7 on phagosomes is not sufficient to enact PL fusion (61). Rather, it requires further interaction with effector proteins to tether the vesicles together to facilitate fusion.

## Securing the Phagosomes to Lysosomes: Tethering Effectors

Tethering effectors, in the form of proteins and protein complexes, are recruited from the cytoplasm to specific membranes designated by small GTPases such as Rab or ADP-ribosylation factor-like protein (Arl) proteins (17). During the tethering step prior to fusion, tethering factors bind to Rab GTPases on the adjacent vesicles to provide an initial physical interaction (62). In addition to their function as linkages between two vicinal membranes, tethers can recruit SNARES as well as the proteins that catalyze the formation of the SNARE complex. Moreover, tethers can strengthen the association amongst fusion-relevant factors at the fusion site. Altogether, tethers ensure fusion fidelity and increase the efficiency of SNARE formation at the docking site.

Rab GTPases modulate fusion of vesicular membranes through their effector proteins. One of the most well-characterized Rab7 effectors is the Rab7-interacting lysosomal protein (RILP) which is found on endosomes, lysosomes, and phagosomes (47, 63). The role of RILP in PL formation lies in its interaction with the dynein-dynactin complex during centripetal migration of phagosomes (47). Further, RILP recruits other Rab effectors involved in vesicle tethering and Rab7 activation. Thus, RILP recruitment is an attractive target for intracellular pathogens to exploit. Indeed, live *Mycobacteria* were found to secrete a Rab7 deactivating factor that could disrupt the interaction between RILP and membrane-bound Rab7 in macrophages (30). This was later determined to be nucleoside diphosphate kinase (Ndk), which enacts GAP activity to lock Rab7 in a GDP-bound state, and permitted survival of *Mycobacteria* within the phagosome (64). While this effect is likely not specific to RILP-Rab7 interactions, it does highlight the importance of maintaining Rab7 in a GTP-bound state to facilitate effector protein function.

Pleckstrin homology domain-containing family M member 1 (Plekhm1) is another Rab7 adaptor that is implicated in modulating PL formation. After the uptake of *Salmonella* by macrophages, Plekhm1 has been shown to bind the multi-subunit HOPS complex which facilitates the delivery of late endosome and lysosomes to *Salmonella*-containing vacuoles, and this is essential to maintain the integrity of these phagosomes for bacterial persistence (65, 66).

The multi-subunit complex HOPS is a Rab7 effector that can function as an upstream Rab7 GEF and a downstream tethering effector (56, 67, 68). Mammalian HOPS is a well-characterized tethering complex that facilitates vesicle-lysosome fusion of multiple degradative pathways, including PL fusion. The complex consists of seven proteins: Vps11, Vps16, Vps18, Vps33A, Vps39, and Vps41 (56, 69, 70). Two subunits appear to be particularly crucial for tethering. Vps39 and Vps41, which are respectively recruited to the HOPs complex by Vps11 and Vps18, can each interact with RILP and this may bridge two RILP molecules on opposing vesicles (**Figure 2B**) (71). Vps41 can also bind directly to Plekhm1 on either lysosomes or phagosomes (66), lysosomal Arl8 (70, 72) and phospoinositide-4-phosphates (PI4P) on phagosomes (73). The capacity for HOPS to bind to multiple partners likely increases the efficiency of HOPS acquisition to the fusion site, improves tethering between vesicles and maintains fusion fidelity (73).

In addition to its function in vesicle tethering, the HOPS complex can promote *trans*-SNARE docking at the PL fusion site and guide the formation of SNARE complexes to promote SNARE-mediated PL fusion (73). The HOPS Vps33 subunit is a member of the SM-family of proteins (74, 75) that can interact directly with SNAREs (76) and is proposed to catalyze SNARE complex assembly. Insights from yeast models suggest a dual-binding capacity of Vps33, where it binds two SNAREs, each from a different membrane, and serve as a base for generating partially-formed SNARE intermediates (24, 25). Bach et al. (77) provided evidence that mammalian Vps33 has a similar function during PL fusion through host-pathogen studies in *Mycobacterium*-infected THP-1-derived macrophages. Protein phosphatase 2A (PtpA), produced by *M. tuberculosis*, can dephosphorylate and inactivate Vps33b, one of the two Vps33 proteins in higher eukaryotes, as evidenced by a reduction in PL fusion after the uptake of PtpA-coated experimental particles by macrophages (77). PL biogenesis was similarly abrogated in Vps33b-silenced macrophages with or without PtpA-coated experimental particles and re-introduction of active Vps33b to the Vps33b-silenced macrophages restored PL fusion. The observation that functional Vps33b is important in PL biogenesis and bacterial clearance is even further supported by the observation that the loss of Vps33b or its binding partner Vps16b in the *Drosophila* Oregon-R phagocytic cell line leads to defects in PL formation (78). Taken together, all seven HOPs subunits are necessary for phagosome maturation.

The HOPS complex is the most extensively characterized Rab7 tethering effector facilitating PL fusion. Since other Rabs have been implicated in PL biogenesis, such as, Rab2 and Rab14, non-Rab7-facilitated HOPS recruitment is possible, or other tethering factors are involved in PL biogenesis, which warrants further investigation. Additional Rab7 adaptors including oxysterol-binding protein-related protein 1L (ORP1L) (79), Vps34/p150 (80) and Rubicon (81) have been characterized in endosome maturation but have yet to be explored in the context of phagosome maturation. Overall, the multifunctional HOPS complex provides a base at the PL fusion site that allows for local SNARE assembly, stabilizing the intermediate complex as the SM protein activity of HOPS chaperones SNARE assembly.

## Fusing the Phagosome and Lysosome Membranes: SNAREs

SNARE proteins compose the core machinery required to fuse phagosomes with lysosomes efficiently. Based on the contributing amino acid to the hydrophobic core in the assembled SNARE complex, SNAREs can be structurally categorized into R-SNAREs or Q-SNAREs (of which can be subclassified into Q_a_-, Q_b_-, and Q_c_-SNAREs) (82). During membrane fusion, an R-SNARE on one membrane forms a transient *trans*-SNARE complex with three Q-SNARES located on the partner membrane (82). The resulting complex consists of an R, Q_a_-, Q_b_-, and Q_c_-SNARE that aligns in a parallel four-helix bundle and fuses the juxtaposed membranes *via* a zipper model (22). Post-fusion, all of the SNAREs are located on the same membrane in a fully assembled *cis*-SNARE complex. The post-fusion *cis*-SNARE complex is then recognized and disassembled by NSF along with its cofactor α-SNAP (27).

Early studies performed in J774 macrophages and cell-free systems demonstrated that PL fusion is dependent on NSF, providing first experimental evidence that this process employs SNARE proteins (83, 84). According to several quantitative proteomic studies, the presence and abundance of different SNAREs found at the phagosome changes depending on the cell-type, stage of maturation and the phagocytosed cargo (11, 85–88). As many as 30 of the 38 known human SNARE proteins have been identified on phagosomes (10), but which of these members function in coordinating late-stage phagosome-lysosome fusion is not well described. Rather, there is a greater understanding of their involvement in phagocytosis, phagosomal trafficking and early phagosome maturation, than during the final stages of maturation. The SNAREs that have thus far been implicated in PL fusion and found to be enriched in lysosomes and phagosomes, include, Syntaxin (Stx) 7, Stx8, vesicle transport through interaction with T-SNAREs 1b (Vti1b), vesicle associated membrane protein 7 (Vamp7) and Vamp8 (37, 39, 89, 90). Initial evidence of the involvement of these SNAREs in PL fusion was provided in a cell-free study wherein SNARE function was inhibited by the addition of the soluble cytosolic domain of various SNAREs (37). These truncated SNARE fragments outcompete their endogenous counterparts for binding with their membrane-bound cognate partners and form a stable SNARE complex that cannot mediate fusion because they lack transmembrane anchors. In the presence of the truncated form of the Q_abc_-SNAREs Stx7, Vti1b and Stx8, and the R-SNAREs Vamp7 and Vamp8, PL fusion was reduced, as evidenced by decreased lumenal mixing between lysosomes and phagosomes (37). Stx7-Vti1b-Stx8 were found to form a stable quaternary complex with the lysosomal R-SNARE Vamp7 or Vamp8, suggesting these may constitute a possible SNARE complex in PL fusion (37). In summary, Stx7-Vti1b-Stx8 may form a ternary Q_abc_-SNARE intermediate on the late phagosome that can then mediate PL fusion together with Vamp7 or Vamp8 on the lysosome, although evidence is lacking to support that these particular SNARE complexes are occurring within living cells to facilitate the fusion of phagosomes with lysosomes.

Interestingly, several investigations into the molecular mechanism underlying gp91^phox^ recruitment to phagosomes have provided insight into PL fusion, and identified synaptosomal-associated protein 23 (Snap23) as a potential Q_bc_-SNARE acting in this process (38, 39, 91). gp91^phox^ is the integral membrane component of nicotinamide adenine dinucleotide phosphate (NADPH) oxidase 2 complex (Nox2) which is responsible for generating the reactive oxygen species (ROS) that facilitate pathogen killing and the modulation of phagosomal proteolysis for antigen processing (92–94). The phagosome accumulates gp91^phox^ predominantly through its fusion with lysosomes (95–97). siRNA-mediated knockdown of Snap23, Stx7, and Vamp8 decreased phagosomal gp91^phox^ recruitment and subsequent ROS production in J774 macrophages, presumably from inhibited PL fusion (39). In corroboration with this observation, silencing Snap23 in primary neutrophils (91) and DCs (38) also inhibited gp91^phox^ trafficking to phagosomes for subsequent ROS production. Whether Snap23 can act as the Q_bc_-SNARE instead of Vti1b-Stx8 in the Stx7-Vti1b-Stx8-Vamp7/8 model described above was supported by immunofluorescence and cellular fractionation experiments which found that Snap23 colocalizes with Stx7 and Vamp8 and can interact with Stx7 in J774 macrophages (38, 39). Moreover, Snap23, Stx7, and Vamp8 can form a stable SNARE complex *in vitro* (39) and knockdown of any of these SNAREs decreases phagosomal acquisition of gp91^phox^ (38, 98). In another study, Vamp8 contributed to the regulation of phagosomal oxidative activity *via* PL fusion in *Leishmania*-infected primary macrophages (99). It is worth noting that Snap23 can also pair with Vamp7 (39), thus Vamp7 and Vamp8 may have redundant roles as a cognate R-SNARE on lysosomes.

Further insight into the function of Snap23 in PL formation was generated from a recent study by Sakurai et al. (40) that reported Snap23 could regulate PL formation based on its phosphorylation status. It was found that the phosphorylation of Snap23 at Ser95 by the protein kinase inhibitor kappa B kinase 2 (IKK2), in response to interferon-γ (IFN-γ) treatment, decreased fusion between phagosomes and late-endosomes/lysosomes in J774 macrophages. Thus, Snap23 is implicated to have an important physiologic role in activated macrophages by delaying phagosome fusion with acidic lysosomes, thereby contributing to the development of a phagosomal environment that favors antigen processing in pro-inflammatory environments (40, 100).

Current evidence suggests that Vamp7/8 on lysosomes, paired with Stx7 and Snap23/Vti1B-Stx8 on late phagosomes, act as the R-, Q_a_- and Q_bc_-SNAREs respectively, to facilitate fusion of these vesicles (**Figure 2B**). However, the promiscuous nature of SNARE proteins likely allows multiple SNARE complex permutations to facilitate the fusion of phagosomes and lysosomes and is reflected in the studies examined (10, 37, 39, 40). Similarly, multiple levels of posttranslational regulation of SNARE proteins beyond Snap23 phosphorylation likely exist to prevent erroneous fusion of these vesicles yet remain to be characterized  (40).

## Other Factors Involved in PL Fusion

It is clear from the number of fusion proteins discussed thus far that PL fusion is a complex process. However, Rabs, tethers and SNAREs are not the only factors involved in this process. The lipid composition of phagosomes, the actin cytoskeleton, the vacuolar-type ATPase (V-ATPase) and calcium signaling play important roles during phagosome maturation. Here, we will only discuss the phosphoinositide lipids and the controversial role of actin and the V-ATPase in PL biogenesis, as calcium signaling has been extensively reviewed elsewhere (101).

### Phosphoinositide Lipids and Their Kinases

Phosphoinositides (PIs) play a critical role in PL formation. These are a family of mono-, bi-, or tri- phosphorylated derivatives of the glycerophospholipid phosphatidylinositol. Phosphorylation of the third, fourth or fifth position of the PI inositol headgroup by their specific lipid kinases generates different PI variants that regulate the actin cytoskeleton, signal transduction and membrane fusion/fission through interactions with their respective effector proteins (102, 103). In the context of PL biogenesis, PI3P and PI4P together with their lipid kinases are the most well-characterized in the fusion of phagosomes and lysosomes (73).

P13P is enriched on early phagosomes and is necessary to bind Rab5 for fusion with early endosomes (5, 104). As the phagosome progresses to the early-to-late-stage transition, PI3P is lost while both PI4P and PI3,5P2 are acquired in tandem with Rab7 (105, 106). PI4P is associated with the recruitment of the HOPS complex (73), whereas the role of PI3,5P2 remains less understood and there is conflicting reports on whether this lipid is essential for PL fusion and the full activation of the phagosomal V-ATPase (107, 108). Reduced PI3,5P2 synthesis in Raw264.7 macrophages abrogated both PL fusion and degradative capacity but had little effect on phagosome acidification, and at least on lysosomes, the V-ATPase was still active (107, 109). However, in lower eukaryotic models and mammalian epithelial cells, PI3,5P2 was required for V-ATPase activity, efficient phagosome fusion with lysosomes and acquisition of a microbicidal phagosomal lumen (108, 110–112). Thus, these discrepancies are likely due to physiological differences between macrophages and other cell models/types. Interestingly, PI3,5P2 has also been implicated in regulating lysosome/phagosome calcium channels in Raw264.7 macrophages (113).

Deciphering the roles of PIs during each step of PL formation within phagocytes is challenging as PIs function in the early steps of phagocytosis, and interference of PIs also affects the maturation of the phagosome upstream of PL fusion (114). Thus, reconstitution of PL fusion in cell-free *in vitro* assays in this scenario provides an advantage over *in vitro* systems since each fusion “subreaction” in PL formation can be manipulated and studied in isolation, without interfering with the early maturation of the phagosome (73, 114). In particular, work by Jeschke and colleagues have shed additional light on the role of PI3P, PI4P and their lipid kinases. As found by Jeschke et al. (114), phagosomes containing opsonized latex beads isolated from J774 macrophages contained PI3P, PI4P, and the class II phosphatidylinositol 4-kinase α (PI4KIIα), one of the four lipid kinases that catalyze the formation of PIs to PI4P (114). Sequestration of PI3P by the mouse hepatocyte growth factor-regulated tyrosine kinase substrate 2xFYVE domain or PI4P by the P4C fragment of *Legionella pneumophila* protein SidC blocked PL formation (115, 116). Further, chemical inhibition of either PI4KIIα or PI3KIII/Vps34 respectively reduced the levels of PI4P or PI3P generated on phagosomes, also impeding PL formation. This corroborates earlier studies, wherein inhibition of PI3KIII/Vps34 in J774 and Raw264.7 macrophages precluded the acquisition of the late endosome/lysosome makers, LAMP1 and lysobisphosphatidic acid, by phagosomes due to a defect in PL fusion (5, 104). Hence, PI3P and PI4P are required for PL fusion in a cell-free system, and this process can be regulated by PI lipid kinases that modulate the levels of PI3P and PI4P generated on phagosomes (114).

Further investigation by Jeschke and Haas (73) into the role of PI3P and PI4P in the steps leading to PL fusion observed that these PIs were involved during the tethering step of membrane fusion. Both PI3P and PI4P were present on phagosomes bound to lysosomes and sequestration of PI4P with P4C inhibited phagosome-to-lysosome binding and hemi-fusion of vesicles, while sequestration of PI3P by the 2xFYVE domain inhibited PL fusion after vesicles had docked (73). Since PIs exert their functions by anchoring PI effector proteins to membranes, it was postulated that PI3P and PI4P were recruiting PL-fusion-relevant proteins to phagosomes and lysosomes (73, 117). Indeed, a lack of available PI3P due to sequestration decreased the presence of the HOPS component Vps41 on phagosomes and lysosomes, and similarly, PI4P sequestration decreased both Vps41 and Arl8 levels. Further, when P4C and 2xFYVE were added to fusion sub-reactions containing isolated phagosomes and lysosomes but in the absence of cytosol, (which contains free PIs), membrane-bound Vps41 and Arl8 were absent, thereby supporting the observation that PI3P and PI4P are required to anchor tethering effectors. In addition to tethering, PI3P and PI4P have an impact on the fusion step of PL biogenesis, and thus may directly or indirectly recruit SNARE proteins (73). Interestingly, Vamp8 can bind directly to PI3P on *Salmonella*-containing vacuoles (118). It is known that Vamp8 binds PI3P to support phagocytosis of *Salmonella* (118), but whether the Vamp8-PI3P interaction is required or able to facilitate SNARE complex formation or PL membrane fusion requires further investigation. PI4P is also a substrate for the formation of PI4,5P2 on late endosomes/phagosomes and lysosomes (119). PI4,5P2 is required for the nucleation of actin around phagosomes, wherein this process has been postulated to promote PL fusion by interacting with SNARE proteins and will be discussed below (119, 120).

The class IA PI3K (PI3KIA), a kinase commonly associated with phagocytosis of large particles (>4 µm) and early endosome-phagosome fusion, has also been implicated in PL fusion (121). Thi and colleagues (121) used THP-1-derived macrophages to report a novel role for the class IA PI3K in PL fusion that depended on its catalytic subunit p110α (121). In p110α knockdown cells, phagocytosis of *M. smegmatis* or experimental particles occurred normally, however, phagosomes displayed impaired procurement of LAMP1 and lysosomal hydrolases due to defective fusion with lysosomes. Interestingly, p110α knockdown did not prevent Rab7 recruitment to phagosomes, nor its subsequent activation, as the recruitment of the Rab7 effectors HOPS and RILP were unaffected. This suggests recruitment of Rab7 and its effectors is insufficient to drive PL fusion alone, and requires the additional action of PI3KIA to mediate the formation of the PL. Moreover, cellular and phagosomal levels of the SNAREs Vamp7 and Vti1b in p110α-silenced macrophages were similar to the control. Hence, in this experimental system, the blockage of PL fusion is not due to a lack of membrane fusion machinery, but an undetermined mechanism. PIK3IA generates PI3,4,5P3 by the phosphorylation of PI4,5P2 (122). PI3,4,5P3 is transiently enriched at the phagocytic cup and is known for its role in actin polymerization upon Fc-γ receptor and complement receptor-mediated phagocytosis (123). With the exception of Fc-γ receptor phagocytosis, PI3,4,5P3 reappears on the maturing phagosome and is implicated in activating a second wave of actin formation important for PL biogenesis (124, 125). Since PI3,4,5P3 levels are decreased upon depletion of p110α (121), it is plausible that this may interfere with PI3,4,5P3-regulated actin polymerization around phagosomes (124).

Overall, host-pathogen studies have unveiled the consequences to bacterial clearance when certain PIs are unavailable for PL fusion while cell-free assays have allowed us better to understand where and when certain PIs are involved in the formation of PLs. From these studies, PI3KIII/Vps34, PI4KIIα and their respective lipid products PI3P and PI4P, as well as PI3KIA, regulate the development of PLs.

### The Actin Cytoskeleton

**Figure 3:**
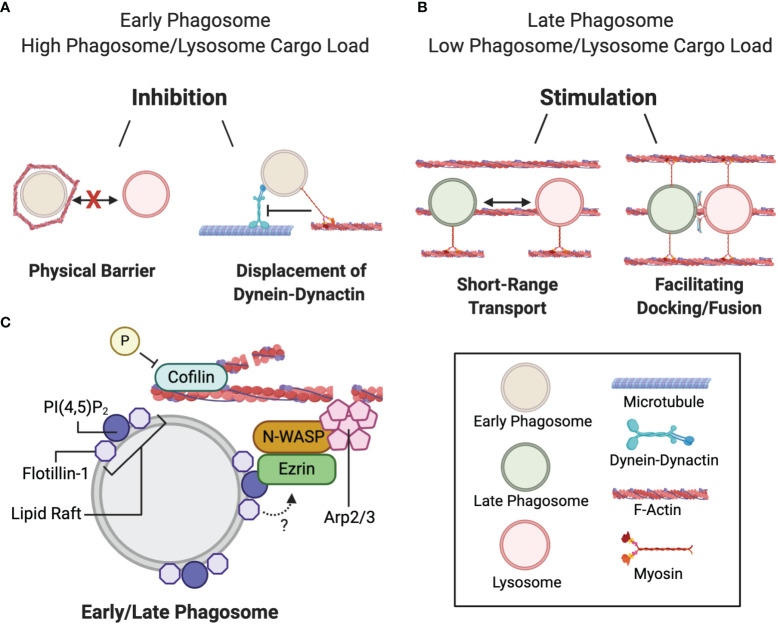
Duality of filamentous actin during phagosome maturation. **(A)** Actin delays phagolysosome biogenesis at the early phagosome maturation stage by: i) transiently assembling around nascent phagosomes when the early phagosome and/or lysosome system is overloaded with cargo thereby blocking phagosome-lysosome contact, or ii) by facilitating myosin-mediated displacement of the dynein-dynactin microtubule motor and preventing minus-end transport. **(B)** Actin stimulates phagolysosome biogenesis by: i) bringing the fusing organelles closer together, or ii) facilitating docking and fusion. **(C)** Actin polymerization at the phagosome is mediated by the ezrin-N-WASP-Arp2/3 complex binding to phagosomal PI(4,5)P_2_ at flotillin-1-associated lipid rafts. Cyclic actin polymerization and depolymerization is mediated by the respective phosphorylation and dephosphorylation of cofilin.

The actin cytoskeleton is a major player in regulating phagocytosis (126, 127) and the fusion/fission of endolysosomes with phagosomes (120, 125, 128–130). Filamentous actin (hereafter referred to as actin) has been observed to transiently polymerize around phagosomes in a process called “actin-flashing”. The function of this phenomena is unclear but has been proposed to prevent PL fusion (125). Numerous reports using phagocytes and cell-free systems have demonstrated that actin coats a subset of purified phagosomes and acts as a physical barrier to PL biogenesis (**Figure 3A**) (125, 131–133). Furthermore, when internalized, certain pathogens, including *Legionella*, *Leishmania*, *and Mycobacteria*, induce the assembly of actin on the early phagosome to prevent phagosomal maturation (133–136). However, conflicting reports propose that actin can have a stimulatory role in membrane fusion, thus lending uncertainty to the role of actin in PL fusion (**Figure 3B**) (120, 128). A plausible explanation is that actin has dual roles in membrane fusion whereby it can both inhibit and induce phagosome-lysosome fusion depending on the maturation state of the phagosome (120). Most studies reporting the inhibitory role of actin limited their investigation to early-stage phagosomes. In particular, transient accumulations of actin were observed to surround a subpopulation of purified immature phagosomes which prevented association with endolysosomal vesicles (125, 137). It was also demonstrated that *de novo* actin assembly on nascent phagosomes increases under cell stress conditions when the phagocytic or endocytic pathway is overloaded with digestive cargo (125). These actin “flashes” repeat in waves to physically block phagosome-lysosome fusion.

Not only can actin act as a physical barrier to fusion, it can also compete with dynein-dynactin-based binding to microtubules at the cellular periphery (**Figure 3A**). Phagosomes can bind to actin, in an ATP-dependent manner, through association with the actin motor protein myosin Va (131). Myosin Va interactions occur at defined regions of the phagosomal membrane, which dissociates the interactions with MT-associated proteins. It has been postulated that transient actin assembly on endolysosomal and phagosomal systems slows phagosome-lysosome fusion when these vesicles contain more cargo than they have the capacity to digest (125). Actomyosin association is a possible complimentary mechanism that can either delay phagosome migration to the perinuclear region to allow them extra time to preprocess internalized phagosomal content, and to not outpace the availability of lysosomes for fusion (131, 137).

In contrast, late-stage phagosomes and endolysosomes have been shown to nucleate actin locally around the phagosome-to-lysosome docking sites (128). Interestingly, numerous actin cytoskeleton-associated machinery regulating actin nucleation were localized around latex bead-containing late-phagosomes, such as ezrin, ﻿neural Wiskott-Aldrich syndrome protein (N-WASP) and the actin-related protein 2/3 (Arp2/3) complex (**Figure 3C**). Ezrin binds to PI4,5P2 on the phagosomal membrane, although it is unclear whether PI4,5P2 is sufficient to recruit ezrin (119, 138). Ezrin has been proposed to recruit N-WASP to the phagosome membrane, which subsequently activates Arp2/3 to initiate actin nucleation. The idea that actin promotes late-stage phagosome maturation was further supported by the observation that downregulating ezrin hindered PL fusion (120). In later studies, cell division control protein 42 homolog (CDC42) of the Rho-family of GTPases was found to be required for the activation of N-WASP (139, 140). The dynamic phosphorylation and dephosphorylation of actin-binding protein cofilin is also required to mediate cyclic actin polymerization and depolymerization that promotes PL fusion (141). Furthermore, flotillin-1, a membrane protein that associates with lipid rafts on late phagosomes is also implicated in actin nucleation since actin predominantly accumulates and polymerizes at these sites (142, 143). Dermine et al. (142) observed decreased flotillin-1 association with phagosomes containing *Leishmania* in J774 macrophages. *Leishmania* expresses a surface glycolipid lipophosphoglycan that impedes PL fusion. Since lipophosphoglycan preferentially anchors to phagosomal lipid rafts, it was proposed that this process may interfere with lipid raft formation, and subsequent flotillin-1 association and actin assembly (142). However, the molecular interactions between flotillin-1 and the other actin polymerizing machinery at phagosomal lipid microdomains were not addressed and remain largely obscure. How actin functions in tandem with membrane fusion machinery—whether by bringing fusing vesicles together *via* myosin motor proteins, or by facilitating the docking and tethering of lysosomes to the phagosome membrane (**Figure 3B**) (120, 128)—is unclear and warrants further investigation.

Interestingly, the same actin nucleation machinery is recruited to early and late phagosomes to exert dualistic stimulatory and inhibitory effects. *Leishmania donovani* promastigotes promote CDC42 retention at phagosomes thereby prolonging phagosomal actin accumulation to evade the host endomembrane system in Raw264.7 macrophages (135). It was recently found that CDC42 is unable to associate with its GAP and GDI in this context, thereby locking it in a GTP-bound state (144, 145). One of these studies demonstrated that *Shigella flexneri* exploits N-WASP and Arp2/3 recruitment to the nascent bacteria-containing vesicles to impede lysosomal fusion. Molecularly, the acyltransferase activity of the type 3 secretion system effector IcsB on CDC42 disrupts its association with its GDI (144), locking CDC42 in a GTP-bound state which retains CDC42 at phagosomes (145). Through this, IcsB clusters N-WASP and Arp2/3 around vesicles to nucleate thick actin coats, which both block the recruitment of fusion machinery and shield the bacterium from the endolysosomes. It is important to note that the studies by Liu et al. (144) and Kühn et al. (145) were conducted in epithelial cells that although are capable of phagocytosis, do not possess the repertoire of surface receptors of professional phagocytes.

In summary, actin networks are thought to have a dual role in phagosome-lysosome fusion depending on how far the phagosome has matured and the cargo load of phagosomes and lysosomes. Actin networks are inhibitory when phagosomes and lysosomes are saturated with digestive cargo by physically preventing early phagosomes from contacting endolysosomes. Actomyosin could potentially compete with dynein-dynactin motor proteins for binding to the phagosome to delay phagosome maturation. In contrast, actin networks can support late phagosome-lysosome fusion, either by bringing vesicles closer together or by facilitating vesicle docking—although the mechanisms by which actin achieves these functions and whether the involvement of actin is cargo-dependent is unclear. Moreover, the spatiotemporal control of actin that enables it to either block or enhance PL fusion remains uncharacterized. Indeed, the role of the actin-myosin network during phagosome maturation is only beginning to be understood, and further work in the area will help garner appreciation of such complex interactions.

### The Vacuolar ATPase

Phagocytes acidify the phagosomal lumen by recruiting the V-ATPase, a multiprotein complex that consists of 14 different subunits (146). The cytosolic V1 domain mediates ATP hydrolysis, whereas the transmembrane V0 domain forms the proton channel (146). Phagosomal V-ATPase is largely considered to be derived from lysosomes; however, other data suggests that phagosomes acquire V-ATPases from multiple sources, including, the trans-Golgi, early endosomes, lysosomes and the plasma membrane (147–149).

Although it is well-known that V-ATPase-mediated phagosomal acidification is essential for antimicrobial defense and efficient digestion/processing of phagocytosed cargo, there is controversy surrounding the role of the V-ATPase in membrane fusion. The first evidence to demonstrate the requirement of the V-ATPase in homotypic vesicle fusion was from a study in yeast (150). Following *trans*-SNARE formation, the V0 domains from apposing vesicles were proposed to complex, leading to a conformational change which allows for lipid mixing and the formation of a fusion pore (150). An acidification-independent role of the V-ATPase in membrane fusion was observed in higher eukaryotes in subsequent studies (151–153).

The role of the V-ATPase in PL fusion was first observed in a zebrafish model, wherein knockdown of the V0 A1 subunit prevented PL fusion in microglial cells (154). Further, inhibiting the macrophage-specific V0 subunit D2 in murine bone marrow-derived macrophages impaired PL fusion and *Salmonella* clearance (155). Interestingly, by immunoprecipitation and glutathione S-transferase affinity isolation assays, subunit D2 was found to form a complex with the SNAREs Stx17 and Vamp8 important in autophagosome-lysosome fusion (155). Whether the V-ATPase could associate with fusion machinery involved in PL fusion was demonstrated in an earlier study in *Mycobacterium*-infected THP-1 macrophages (156). As mentioned above, the *M. tuberculosis* effector PtpA dephosphorylates the SM protein Vps33B to prevent PL fusion (77). Wong et al. (156) demonstrated that PtpA directly binds to the V1 subunit H to: i) block the trafficking of this subunit to the mycobacterial phagosome and ii) block its interaction with Vps33B (156). Moreover, this binding step is a prerequisite to dephosphorylate Vps33B. When macrophages were challenged with *ptpA* knockout strains, subunit H could localize to phagosomes and recruit Vps33B. Contrary to these findings implicating the V-ATPase in PL biogenesis, other reports dispute this contribution.

In mouse peritoneal macrophages, the loss of the V0 subunit A3 did not block PL formation during Fc-γ receptor phagocytosis of IgG-opsonized latex beads—phagosomes could acquire lysosomal characteristics including the mature forms of lysosomal cathepsins, and late-phagosome/lysosome markers Rab7 and Lamp2 (157). In corroboration with these findings, mouse bone marrow-derived macrophages deficient in the V0 subunits A1-3 had unaltered microbicidal activity since these cells could clear avirulent strains of *Escherichia coli* and *Listeria innocua* as efficiently as the control (158). Interestingly, through pulse-chase experiments with latex beads, the rate of PL fusion was elevated upon the loss of the V0 subunits A1-3.

Thus, whether the V-ATPase enhances PL fusion is still being debated. Different phagocytic cargoes and/or cell models leading to slightly altered maturation pathways may explain discrepancies in the works described. Moreover, it appears that specific subunits of the V-ATPase (V0 subunit D2 and V1 subunit H) have more important roles in supporting PL fusion than others (V0 subunits A1-3).

## Autophagy to the Rescue: Supporting Phagocyte Function in the Context of PL Fusion

As noted earlier, several pathogens have evolved mechanisms to subvert canonical maturation of the phagosome. In response, alternative mechanisms to facilitate PL fusion have coevolved in professional phagocytes. Macroautophagy, or bulk degradation autophagy (herein referred to as autophagy), has been established as a fundamental homeostatic pathway that affects innate and adaptive immunity (159, 160). Similar to the phagocytic pathway for exogenous cargoes, autophagy delivers endogenous material to lysosomes for degradation. Low levels of basal autophagy is essential for all cells to clear unwanted cytosolic material and maintain cellular homeostasis (161–163). In phagocytes, autophagy is upregulated by several stress signals, including pro-inflammatory cytokines  (164), TLR signaling (165), starvation (166), and microbial infection  (167). The role of autophagy in infection, inflammation, and adaptive immunity has received increasing attention over the past 2 decades. Germane to this review, components of the autophagic pathway have been implicated in PL fusion—proposed as an alternate pathway to overcome pathogen inhibition of canonical phagosomal maturation and enhancing the presentation of exogenous antigens. In the following section, we will discuss how the autophagic pathway complements the phagocytic pathway with an emphasis on PL fusion.

### Microbial Clearance

The best-understood pathways by which autophagy augments microbicidal capabilities of phagocytes are xenophagy (168) and ﻿microtubule-associated protein A1/B1-light chain 3 (LC3)-associated phagocytosis (LAP) (169–171). Xenophagy is the selective degradation of cytosolic pathogens or pathogen-containing vesicles that have been marked with ubiquitin through autophagic mechanisms (172–174). In contrast, LAP is a non-canonical form of autophagy that involves phagocytosis and uses some components of the autophagic pathway to create a more robust phagolysosome (169–171).

#### Xenophagy

**Figure 4:**
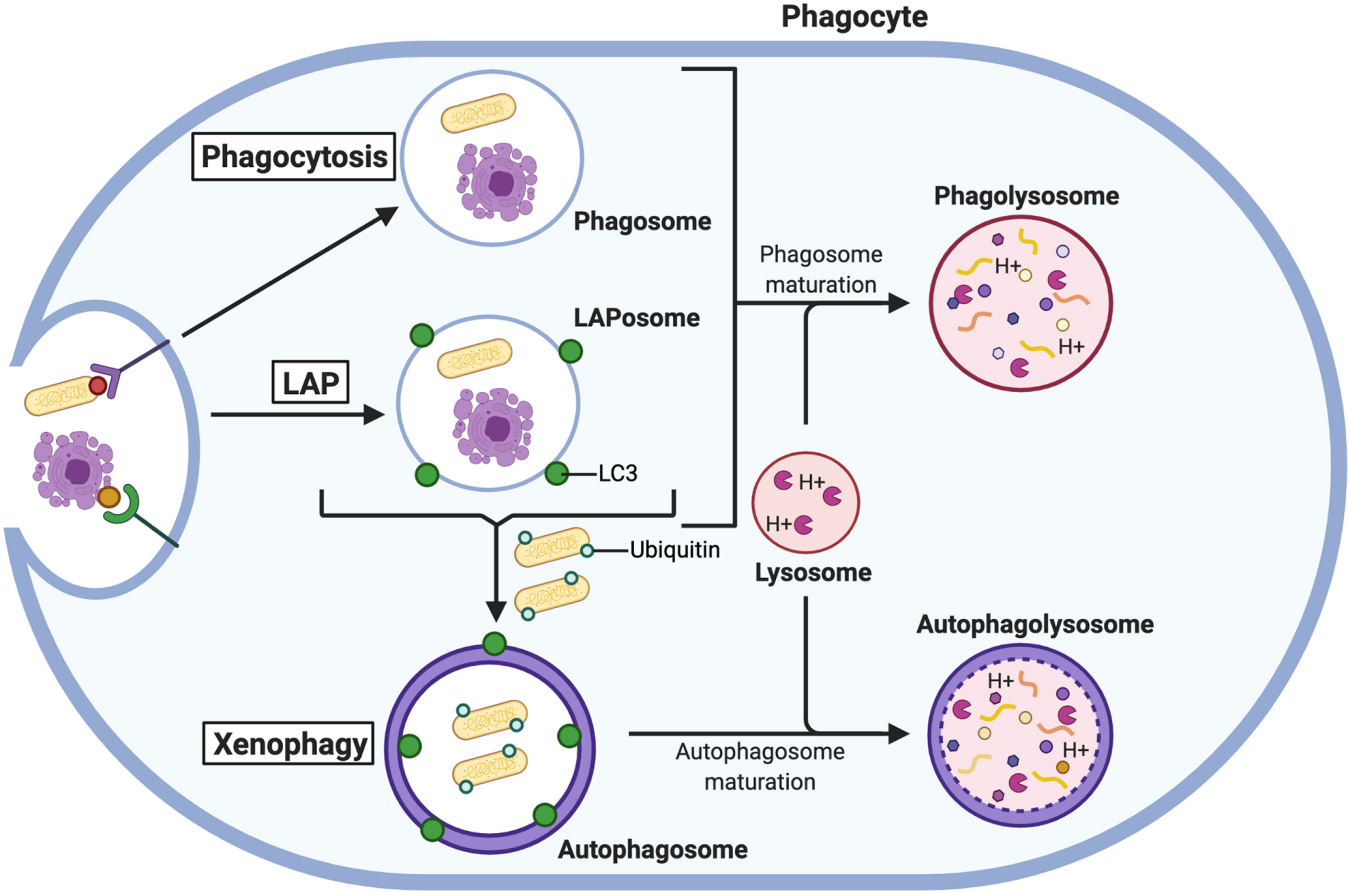
The autophagic response aids the phagocytic pathway. Phagocytosed microbes and apoptotic cells are degraded in PLs formed from the fusion of lysosomes with phagosomes. Under certain conditions, LC3 is recruited to phagosomes for LAP to create more robust PLs. Microbes that escape PL-mediated degradation are ubiquitinated in the cytosol and degraded in autophagolysosomes *via* xenophagy.

Several intracellular pathogens have developed mechanisms to avoid PL-mediated destruction, and autophagy can be deployed to assist in their elimination (**Figure 4**). *M. tuberculosis* is well known to inhibit phagosomal maturation, but induction of autophagy is sufficient to eliminate the pathogen *via* autophagic consumption of the arrested bacteria-containing phagosome  (175). The parasite *Toxoplasma gondii* can survive within macrophages by residing in vacuoles that avoid fusion with lysosomes (176). However, when macrophages are activated by a T cell ligand, cluster of differentiation 40 (CD40), xenophagy is induced to restrict *T. gondii* growth (177, 178). Alternatively, when activated by IFN-γ, macrophages use some components of the autophagic machinery to recruit members of the immunity-related GTPase (IRG) family to mediate the elimination of these parasites in a process that is not fully understood (179, 180). However, evidence from *Mycobacteria*-containing macrophages suggests that IRGs may promote the fusion of phagosomes to lysosomes since Irgm1 has been observed to interact with SNAP-associated protein (Snapin), which binds to SNAREs to mediate vesicular fusion (181, 182).

Although xenophagy functions as a second line of defense against pathogens that overcome PL-mediated killing in phagocytes, some pathogens have further evolved to evade xenophagic activity by inhibiting the autophagic response. For example, the virulence factors of *M. tuberculosis* early secretory antigenic target 6 (ESAT6) and secreted acid phosphatase M (SapM), block autophagosome-lysosome fusion in Raw264.7 macrophage-like cells; ESAT6 stimulates the negative regulator of autophagy mammalian target of rapamycin (MTOR), and SapM hydrolyzes PI3P to prevent Rab7 recruitment to phagosomes and autophagosomes (183–186). In another study using Raw264.7 cells, *L. monocytogenes* was found to hide from autophagic recognition through its virulence factor Inlk (187).

In summary, while xenophagy often serves as an effective second defense to pathogen PL arrest or escape in many cases, some well-adapted pathogens have evolved virulence factors that target these autophagic pathways.

#### LC3-Associated Phagocytosis

Autophagy can also augment the antimicrobial activity of phagocytes by supporting increased PL fusion through LAP (**Figure 4**). The process of LAP requires several members of the canonical autophagic machinery, namely autophagy-related (Atg) 5, Atg7, Atg12, Atg16L, Beclin1, and Vps34 (169), as well as Rubicon and ROS production by Nox2 (171, 188). Despite sharing components, LAP and autophagy are functionally distinct processes that are differentially regulated. For example, Rubicon inhibits autophagy but is required for efficient LAP (189). LAP can be stimulated when internalized particles activate certain surface receptors of phagocytes, including TLRs, Fc receptors, Dectin-1, and the apoptotic cell receptor T cell immunoglobin and mucin domain containing 4 (TIM4) (169–171). When these receptors are engaged, phagosomes recruit LC3 (now referred to as LAPosomes) which promotes rapid fusion with lysosomes and enhances cargo degradation (169, 188). Through LAP, phagocytes are better equipped to deal with microbial infection by bacteria and fungi. For example, PL fusion with phagosomes containing the bacteria *L. monocytogenes* (190) and *Legionella dumoffi* (191), and the fungi *S. cerevisiae* (169) and *A. fumigatus* (188) is promoted by LAP [reviewed by (192)]. In the absence of LC3 recruitment to phagosomes, macrophages could not efficiently kill these pathogens and were more susceptible to sustained infection.

It is unsurprising then that pathogens have also evolved strategies to avoid targeting by LAP. Some prominent examples are CpsA production by *M. tuberculosis* (193), surface metalloprotease GP63 production by *Leishmania major* (194), and melanin production by *A. fumigatus* (195), which all function to evade LAP by preventing the Nox2-mediated ROS production crucial for LC3-recruitment [reviewed by (192)].

It is worth noting that LAP is not the only process that can enhance PL fusion. Enhanced PL fusion was observed despite the absence of Nox2-mediated LC3 recruitment to phagosomes containing IgG-opsonized zymosan and sheep erythrocytes in macrophages (196). This suggests that under certain conditions, proteins other than those belonging to the autophagic pathway such as members of the IRG family mentioned above (181, 182), can also enhance membrane fusion. Alternatively, it has been recently demonstrated that the mechanical stress caused by the growth of some intraphagosomal pathogens can induce lysosome recruitment and fusion with the phagosome as a means to increase phagosomal surface area and maintain membrane integrity (197). Further, LAP has a dual function in antigen presentation depending on the cell type. LAP stimulates rapid PL fusion in murine macrophages, favoring cargo degradation over antigen presentation (169). In contrast, LAP delays recruitment of lysosomes in human macrophages and DCs to stabilize substrates for major histocompatibility complex class II (MHCII)-mediated presentation to the adaptive immunity (198). Thus, LC3-mediated phagosome maturation is host- and condition-dependent and is a dynamic process that is influenced by the activation of phagocytes.

How LAP promotes or inhibits PL fusion at the molecular level is only beginning to be discerned. One plausible mechanism was seen in neuronal cells, where LC3 binding of RILP to autophagosomes facilitated the recruitment of the dynein-dynactin motor complex for subsequent centripetal movement to the lysosome-rich perinuclear region (199). It would be interesting to investigate whether phagosomal LC3 can recruit RILP-dynein-dynactin to facilitate phagosome trafficking in a similar manner in phagocytes, particularly in the context of LAP. Another possibility is that LC3 plays a role in recruiting machinery to the phagosome that can assist in tethering lysosomes to phagosomes (192). Plekhm1 can be recruited by LC3 to autophagosomes in HeLa cells (200). By extension, LC3-recruited Plekhm1 on phagosomes could serve to tether HOPS- and RILP-containing vesicles during PL formation. Additionally, the LC3 isoforms γ-aminobutyric acid receptor-associated protein (Gabarap) and Gabarap-like 2 can recruit PI4KIIα (201), which generates PI4P on phagosomes and lysosomes to promote HOPS recruitment (114, 202). However, direct evidence to support these hypotheses in phagocyte LAPosomes has yet to be substantiated and awaits further investigation.

### Homeostasis and Inflammation Dampening

Rapid and efficient clearance of apoptotic cells by phagocytes is critical for the regulation of tissue and immune homeostasis. Professional phagocytes are recruited by chemo-attractants released by apoptotic cells, and the “eat-me” cell-surface ligands on the dying cells mark them for phagocytotic engulfment by efferocytosis. It is now well-established that autophagy has a complex relationship with apoptosis in that autophagy can either be preventative or facilitative in programmed cell death [discussed comprehensively by (203)]. Most recently, LAP has been established as a crucial process in efferocytosis (**Figure 4**). Disrupting LAP in macrophages during TIM4-mediated engulfment delays degradation of apoptotic and necrotic cells in phagosomes (204). In the absence of LAP (created by deleting key autophagic genes), apoptotic bodies accumulate in phagosomes but are not digested due to a decrease in lysosomal fusion (205). Moreover, the accumulation in apoptotic cargo leads to secondary necrosis in macrophages. This was also observed in another study by Zhou et al. (206) in which autophagy had a cytoprotective effect on macrophages that phagocytosed apoptotic cells. Silencing a key autophagic protein, Beclin1, in macrophages displayed increased apoptotic cell cargo resulting in decreased viability and survival, and macrophage rupturing (206). When stimulated with apoptotic cells, macrophages deficient in LAP due to genetic knockout of Rubicon or Nox2 produced higher levels of pro-inflammatory cytokines (interleukin-(IL)-1β, IL-6, CXCL10) and lower levels of anti-inflammatory cytokines (IL-10) compared to LAP-sufficient cells (205). This increase in pro-inflammatory signals translates to autoimmunity and the development of systemic lupus erythematosus-like disease in *in vivo* mouse models when LAP is not functioning to clear apoptotic cells (205). Hence, LAP-enhanced PL fusion is fundamental to regulate pro-inflammatory and pro-death signals.

In summary, autophagy and phagocytosis are two complementary pathways that cooperate in PL-mediated elimination of dangerous exogenous and endogenous material to regulate infection and homeostasis. Canonical autophagic machinery is employed to either supplement or support phagosome maturation or to conduct microbicidal and homeostatic functions. Induction of the autophagic machinery enhances PL biogenesis *via* LAP, although the molecular mechanism has not yet been fully characterized. Current evidence suggests LC3-recruitment of phagosome trafficking and fusion machinery, yet this warrants further investigation.

## Concluding Remarks

Here we have summarized recent findings regarding phagosome-lysosome fusion. The Rab GTPases Rab7, Rab2 and Rab14 participate in tethering phagosomes and lysosomes albeit through mechanisms that are less known for Rab2 and Rab14. Rab7 recruits tethering effectors, whereby the individual subunits of the HOPS complex give it the multifunctional capacity to serve as a Rab GEF, a tether and an SM protein that catalyzes SNARE assembly. The SNAREs Snap23, Stx7, Vamp8, Vti1b, and Stx8 have been shown to assemble into specific complexes to execute PL fusion. While the Rabs, tethering factors and SNAREs involved in PL biogenesis are best known, we know less about how phagosomal lipids, the actin network and the V-ATPase regulate PL fusion. PI3P and PI4P have been implicated to interact with fusion machinery, whereas PI4,5P2 is involved in actin nucleation, which exhibits dual function to either support or inhibit PL fusion. PI3,5P2 is required for V-ATPase activation and PL fusion in lower eukaryotes but this has yet to be shown in mammalian phagocytes. Acidification-independent roles of the V-ATPase in fusion is still under contention but some evidence suggests the involvement of specific subunits. Lastly, the autophagic pathway can bolster the phagocytic pathway through LAP-enhanced PL fusion, or xenophagy-mediated protection of phagocyte integrity.

Although there have been great advances in understanding the proceedings of the molecular events that mediate PL biogenesis, we have only scratched the surface in delineating the full extent of all the participating molecules and modulating signals. For those that have been identified, little is known regarding their spatiotemporal regulation. The greatest caveat to the existing studies that have furthered our understanding of the aforementioned fusion machinery is that they have been performed extensively in *in vitro* cell-free models or phagocyte-like cell lines which limits our ability to confidently extrapolate to primary phagocytes. To this degree, few studies have investigated the molecules that control phagosome-lysosome fusion in live primary phagocytes, generating a gap between experimental evidence and functional relevance to the management of infection and disease. For studies that have employed primary cell models, it is unclear whether the fusion molecules recruited to phagosomes is consistent for all cargo types. Phagocytic uptake of macromolecular cargos can be mediated by several cell-surface receptors that activate different signaling pathways based on the type of cargo. The activation/inflammation status of these cells can influence the rate and magnitude of the dynamic recruitment of fusion-mediating proteins. Even the type of phagocyte and particular species from which cells are sourced can influence which particular fusion proteins are involved. Thus, further studies are required to more comprehensively characterize this critical and often underappreciated aspect of phagocyte biology.

Phagosome fusion with lysosomes is crucial to the functional outcome of phagocytosis and efferocytosis. Impaired lysosome fusion—and by extension, cargo degradation—disrupts the immune response to microbial and apoptotic cell clearance, and can underlie autoimmune and inflammatory disorders, and persistence of infection. Deciphering the mechanisms that drive and regulate the fusion of phagosomes with lysosomes will not only contribute to the greater knowledge of the pathogenesis of many diseases, but also contribute to our understanding of health, growth, and homeostasis.

## Author Contributions

JN wrote the manuscript with support from RY. All authors contributed to the article and approved the submitted version.

## Conflict of Interest

The authors declare that the research was conducted in the absence of any commercial or financial relationships that could be construed as a potential conflict of interest.

## Abbreviations

Arl, ADP-ribosylation factor-like protein; Arp2/3, actin-related protein 2/3; Atg, autophagy-related; CDC42, cell division control protein 42 homolog; DC, dendritic cell; ESAT6, early secretory antigenic target 6; Gabarap, γ-aminobutyric acid receptor-associated protein; GAP, GTPase-activating protein; GDI, guanine dissociation inhibitor; GEF, guanine exchange factor; HOPS, homotypic fusion and vacuole sorting; IFN-γ, interferon-γ; IKK2, inhibitor kappa B kinase 2; IL, interleukin; IRG, immunity-related GTPase; LAP, LC3-associated phagocytosis; LC3, microtubule-associated protein A1/B1-light chain 3; MAPK, p38α-mitogen-activated protein kinase; MHCII, major histocompatibility complex class II; MT, microtubule; N-WASP, Wiskott-Aldrich syndrome protein; NADPH, nicotinamide adenine dinucleotide phosphate; Ndk, nucleoside diphosphate kinase; Nox2, NADPH oxidase 2 complex; NRBF2, nuclear receptor binding factor 2; NSF, N-ethylmaleimide-sensitive factor; ORP1L, oxysterol-binding protein-related protein 1L; PI, phosphoinositide; PI3K, phosphoinositide 3-kinase; PI3KIA, class IA PI3K; PI3KIII, class III phosphoinositide 3-kinase; PI4KIIα, class II phosphatidylinositol 4-kinase α; PI4P, phospoinositide-4-phosphate; PL, phagolysosome; Plekhm1, pleckstrin homology domain-containing family M member 1; PtpA, protein phosphatase 2A; RILP, Rab7-interacting lysosomal protein; ROS, reactive oxygen species; SapM, secreted acid phosphatase M; SM, Sec1/Munc18; SNAP, soluble N-ethylmaleimide-sensitive fusion factor attachment protein; Snap23, synaptosomal-associated protein 23; Snapin, SNAP-associated protein; SNARE, soluble NSF attachment protein receptor; Snx10, sorting nexin 10; Stx, syntaxin; TIM4, T cell immunoglobin and mucin domain containing 4; TLR, toll-like receptor; V-ATPase, vacuolar-type ATPase; Vamp, vesicle associated membrane protein; Vps, vacuolar protein sorting; Vti1b, vesicle transport though interaction with T-SNAREs 1b.
