# Supplementary material for: Better Together: Current Insights Into Phagosome-Lysosome Fusion
Source: Front Immunol. 2021 Feb 25;12:636078. doi: 10.3389/fimmu.2021.636078 (PMC7946854; doi:10.3389/fimmu.2021.636078)
Supplement: Supplementary file 1 [file Table_1.docx]

Supplementary Material

**Supplementary Table 1.** Major fusion machinery and their roles in phagolysosome biogenesis.

| **Fusion Protein** | **Cell Model** | **Phagocytic Cargo-Used** | **Role in PL Fusion** | **Reference** |
| --- | --- | --- | --- | --- |
| Actin Nucleation Machinery   - Ezrin - CDC42 - N-WASP - Arp2/3 | J774 and Raw264.7 macrophages | Avidin-conjugated latex beads | Ezrin binds to PI4,5P2 on phagosomes and recruits N-WASP, whereby it is activated by CDC42. N-WASP activates Arp2/3, which initiates actin nucleation required for PL fusion. | (1-4) |
| Cofilin | Primary murine macrophages | *Legionella pneumophila* | Mediates cyclic actin polymerization and depolymerization | (5) |
| Flotillin | J774 macrophages | Non-opsonised latex beads | Promotes actin nucleation at phagosomal lipid rafts through an undescribed mechanism. | (6, 7) |
| HOPS complex | *Caenorhabditis elegans*, primary murine macrophages; cell-free system; yeast, THP-1 macrophages, *Drosophila melanogaster* | Apoptotic somatic and germ cells, *Coxiella burnetii*; avidin-conjugated latex beads; *Mycobacterium tuberculosis* | Rab7 GEF; tethers phagosomes to lysosomes; SM activity of Vps33b guides SNARE assembly at the PL fusion site. | (8); (9, 10); (11-14) |
| Irgm1 | Primary murine macrophages | *Mycobacterium tuberculosis* | Binds to the SNARE adaptor Snapin, which interacts with SNAREs to promote PL fusion. | (15, 16) |
| PI3KIA | THP-1 macrophages; J774 macrophages | *Mycobacterium* *smegmatis*, serum-albumin-conjugated magnetic beads; avidin-conjugated latex beads | Is required for PL fusion through an unknown mechanism. | (3, 17, 18) |
| PI3KIII/Vps34 | Cell-free system, J774 and Raw264.7 macrophages | Avidin-conjugated latex beads*, Mycobacterium* lipid-conjugated latex beads, IgG-opsonized erythrocytes and latex beads | Generates PI3P on early endosomes/phagosomes and late phagosomes. PI3P is required for PL fusion. | (19-21) |
| PI3P | Cell-free system; HeLa cells | Avidin-conjugated latex beads | Involved in PL tethering through the interaction with the HOPS complex and Arl8; and possibly in membrane fusion through Vamp8 interaction. | (10, 21); (22) |
| PI3,4,5P3 | Raw264.7 macrophages | C3bi-coated sheep erythrocytes | Implicated in actin polymerization around maturing phagosomes. | (23) |
| PI3,5P2 | Raw264.7 macrophages | IgG-opsonized latex beads | Involved in PL fusion through an unknown mechanism—possibly by regulating lysosome/phagosome calcium signalling important in PL fusion | (24, 25) |
| PI4,5P2 | J774 macrophages | Avidin-conjugated latex beads | Binds to Ezrin which is required for actin nucleation. | (1-3) |
| PI4KII$\alpha$ | Cell-free system | Avidin-conjugated latex beads | Generates PI4P on late endosomes/ phagosomes and lysosomes. PI4P is required for PL fusion. | (21) |
| PI4P | Cell-free system, Raw264.7 macrophages; J774 macrophages | Avidin-conjugated latex beads, *Legionella* *pneumophila*; avidin-conjugated latex beads | Involved in PL tethering through interaction with the HOPS complex and Arl8; substrate for the formation of PI4,5P2 on phagosomes required for actin nucleation. | (10, 21, 26); (1) |
| Plekhm1 | HeLa cells, Raw264.7 macrophages | *Salmonella typhimurium* | Rab7 adaptor. Interacts with the HOPS complex which facilitates the delivery of lysosomes to phagosomes. | (27, 28) |
| Rab14 | *C. elegans*, Raw264.7 and J774 macrophages | Apoptotic somatic and germ cells, *Candida albicans* | Traffics lysosomes to phagosomes through an undescribed mechanism. | (29, 30) |
| Rab2 | *C. elegans* | Apoptotic somatic and germ cells | Traffics lysosomes to phagosomes through an undescribed mechanism. | (29) |
| Rab7 | J774 macrophages, Raw264.7 macrophages, HeLa cells | Mycobacterium tuberculosis, *Coxiella burnetii*, IgG-opsonized sheep erythrocytes and polystyrene beads, *Salmonella typhimurium* | Recruits tethering effectors RILP, HOPS and Plekhm1 to the phagosome/lysosome. | (8, 27, 28, 31, 32) |
| RILP | Raw264.7 and J774 macrophages | IgG-opsonized sheep erythrocytes and polystyrene beads; avidin-conjugated latex beads, *Salmonella typhimurium* | Rab7 adaptor. Binds to the dynein-dynactin complex for phagosome trafficking; interacts with the HOPS complex which is involved in late phagosome maturation. | (31); (9, 27) |
| Snap23 | J774 macrophages, primary murine dendritic cells | IgG-opsonized zymosan | Q_bc_-SNARE on the plasma membrane and phagosomes. Forms a stable complex with Stx7-Vamp7/8 to execute PL fusion. Phosphorylation at Ser95 blocks PL fusion. | (33-36) |
| Stx7 | Cell-free system, J774 macrophages | IgG-opsonized sheep erythrocytes, *Escherichia coli*, IgG-opsonized zymosan | Q_a_-SNARE on late endosomes/lysosomes and phagosomes. Forms a stable SNARE complex with Snap23-Vamp7/8 or Vti1b-Stx8-Vamp7/8 to execute PL fusion. | (35, 37, 38) |
| Stx8 | Cell-free system | *Escherichia coli* | Q_c_-SNARE on late endosomes/lysosomes and phagosomes. Forms a stable SNARE complex with Stx7-Vti1b-Vamp7/8 to execute PL fusion. | (37) |
| Vamp7 | Cell-free system, J774 macrophages | *Escherichia coli*, IgG-opsonized zymosan | R-SNARE on late endosomes/lysosomes. Forms a stable SNARE complex with Stx7-Snap23 or Stx7-Vti1B-Stx8 to execute PL fusion. | (35, 37) |
| Vamp8 | Cell-free system, J774 macrophages, primary murine dendritic cells, primary murine macrophages | *Escherichia coli*, IgG-opsonized zymosan, *Leishmania donovani* | R-SNARE on late endosomes/lysosomes. Forms a stable SNARE complex with Stx7-Snap23 or Stx7-Vti1b-Stx8 to execute PL fusion. | (33, 37, 39) |
| V-ATPase subunits   - V0 A1-3 - V0 D2 - V1 H | Primary murine macrophages; THP-1 macrophages | IgG-opsonized latex beads, *Escherichia coli* and *Listeria innocua*; *Salmonella typhimurium*; *Mycobacterium tuberculosis* | The V0 subunits A1-3 are not involved in PL fusion.  The V0 subunit D2 is involved in PL fusion through an unknown mechanism.  The V1 subunit H recruits Vps33B to phagosomes through direct binding. | (40, 41); (42); (43) |
| Vti1b | Cell-free system | *Escherichia coli* | Q_b_-SNARE on late endosomes/lysosomes and phagosomes. Forms a stable SNARE complex with Stx7-Stx8-Vamp7/8 to execute PL fusion. | (37) |

**References**

1. Defacque H, Bos E, Garvalov B, Barret C, Roy C, Mangeat P, et al. Phosphoinositides regulate membrane-dependent actin assembly by latex bead phagosomes. *Mol Biol Cell* (2002) 13(4):1190-202. Epub 2002/04/16. doi: 10.1091/mbc.01-06-0314. PubMed PMID: 11950931; PubMed Central PMCID: PMCPMC102261.

2. Defacque H, Egeberg M, Habermann A, Diakonova M, Roy C, Mangeat P, et al. Involvement of ezrin/moesin in de novo actin assembly on phagosomal membranes. *EMBO J* (2000) 19(2):199-212. Epub 2000/01/19. doi: 10.1093/emboj/19.2.199. PubMed PMID: 10637224; PubMed Central PMCID: PMCPMC305554.

3. Marion S, Hoffmann E, Holzer D, Le Clainche C, Martin M, Sachse M, et al. Ezrin promotes actin assembly at the phagosome membrane and regulates phago-lysosomal fusion. *Traffic* (2011) 12(4):421-37. Epub 2011/01/08. doi: 10.1111/j.1600-0854.2011.01158.x. PubMed PMID: 21210911.

4. Ho HY, Rohatgi R, Lebensohn AM, Le M, Li J, Gygi SP, et al. Toca-1 mediates Cdc42-dependent actin nucleation by activating the N-WASP-WIP complex. *Cell* (2004) 118(2):203-16. Epub 2004/07/21. doi: 10.1016/j.cell.2004.06.027. PubMed PMID: 15260990.

5. Akhter A, Caution K, Abu Khweek A, Tazi M, Abdulrahman BA, Abdelaziz DH, et al. Caspase-11 promotes the fusion of phagosomes harboring pathogenic bacteria with lysosomes by modulating actin polymerization. *Immunity* (2012) 37(1):35-47. Epub 2012/06/05. doi: 10.1016/j.immuni.2012.05.001. PubMed PMID: 22658523; PubMed Central PMCID: PMCPMC3408798.

6. Dermine JF, Duclos S, Garin J, St-Louis F, Rea S, Parton RG, et al. Flotillin-1-enriched lipid raft domains accumulate on maturing phagosomes. *J Biol Chem* (2001) 276(21):18507-12. Epub 2001/03/30. doi: 10.1074/jbc.M101113200. PubMed PMID: 11279173.

7. Rozelle AL, Machesky LM, Yamamoto M, Driessens MH, Insall RH, Roth MG, et al. Phosphatidylinositol 4,5-bisphosphate induces actin-based movement of raft-enriched vesicles through WASP-Arp2/3. *Curr Biol* (2000) 10(6):311-20. Epub 2000/04/04. doi: 10.1016/s0960-9822(00)00384-5. PubMed PMID: 10744973.

8. Barry AO, Boucherit N, Mottola G, Vadovic P, Trouplin V, Soubeyran P, et al. Impaired stimulation of p38alpha-MAPK/Vps41-HOPS by LPS from pathogenic Coxiella burnetii prevents trafficking to microbicidal phagolysosomes. *Cell Host Microbe* (2012) 12(6):751-63. Epub 2012/12/19. doi: 10.1016/j.chom.2012.10.015. PubMed PMID: 23245320.

9. van der Kant R, Jonker CT, Wijdeven RH, Bakker J, Janssen L, Klumperman J, et al. Characterization of the Mammalian CORVET and HOPS Complexes and Their Modular Restructuring for Endosome Specificity. *J Biol Chem* (2015) 290(51):30280-90. Epub 2015/10/16. doi: 10.1074/jbc.M115.688440. PubMed PMID: 26463206; PubMed Central PMCID: PMCPMC4683254.

10. Jeschke A, Haas A. Sequential actions of phosphatidylinositol phosphates regulate phagosome-lysosome fusion. *Mol Biol Cell* (2018) 29(4):452-65. Epub 2017/12/15. doi: 10.1091/mbc.E17-07-0464. PubMed PMID: 29237821; PubMed Central PMCID: PMCPMC6014173.

11. Song H, Orr AS, Lee M, Harner ME, Wickner WT. HOPS recognizes each SNARE, assembling ternary trans-complexes for rapid fusion upon engagement with the 4th SNARE. *Elife* (2020) 9. Epub 2020/01/22. doi: 10.7554/eLife.53559. PubMed PMID: 31961324; PubMed Central PMCID: PMCPMC6994237.

12. Baker RW, Jeffrey PD, Zick M, Phillips BP, Wickner WT, Hughson FM. A direct role for the Sec1/Munc18-family protein Vps33 as a template for SNARE assembly. *Science* (2015) 349(6252):1111-4. Epub 2015/09/05. doi: 10.1126/science.aac7906. PubMed PMID: 26339030; PubMed Central PMCID: PMCPMC4727825.

13. Akbar MA, Tracy C, Kahr WH, Kramer H. The full-of-bacteria gene is required for phagosome maturation during immune defense in Drosophila. *J Cell Biol* (2011) 192(3):383-90. Epub 2011/02/02. doi: 10.1083/jcb.201008119. PubMed PMID: 21282466; PubMed Central PMCID: PMCPMC3101095.

14. Bach H, Papavinasasundaram KG, Wong D, Hmama Z, Av-Gay Y. Mycobacterium tuberculosis virulence is mediated by PtpA dephosphorylation of human vacuolar protein sorting 33B. *Cell Host Microbe* (2008) 3(5):316-22. Epub 2008/05/14. doi: 10.1016/j.chom.2008.03.008. PubMed PMID: 18474358.

15. Tiwari S, Choi HP, Matsuzawa T, Pypaert M, MacMicking JD. Targeting of the GTPase Irgm1 to the phagosomal membrane via PtdIns(3,4)P(2) and PtdIns(3,4,5)P(3) promotes immunity to mycobacteria. *Nat Immunol* (2009) 10(8):907-17. Epub 2009/07/22. doi: 10.1038/ni.1759. PubMed PMID: 19620982; PubMed Central PMCID: PMCPMC2715447.

16. MacMicking JD, Taylor GA, McKinney JD. Immune control of tuberculosis by IFN-gamma-inducible LRG-47. *Science* (2003) 302(5645):654-9. Epub 2003/10/25. doi: 10.1126/science.1088063. PubMed PMID: 14576437.

17. Kjeken R, Egeberg M, Habermann A, Kuehnel M, Peyron P, Floetenmeyer M, et al. Fusion between phagosomes, early and late endosomes: a role for actin in fusion between late, but not early endocytic organelles. *Mol Biol Cell* (2004) 15(1):345-58. Epub 2003/11/18. doi: 10.1091/mbc.e03-05-0334. PubMed PMID: 14617814; PubMed Central PMCID: PMCPMC307552.

18. Thi EP, Lambertz U, Reiner NE. Class IA phosphatidylinositol 3-kinase p110alpha regulates phagosome maturation. *PLoS One* (2012) 7(8):e43668. Epub 2012/08/29. doi: 10.1371/journal.pone.0043668. PubMed PMID: 22928013; PubMed Central PMCID: PMCPMC3425514.

19. Fratti RA, Backer JM, Gruenberg J, Corvera S, Deretic V. Role of phosphatidylinositol 3-kinase and Rab5 effectors in phagosomal biogenesis and mycobacterial phagosome maturation arrest. *J Cell Biol* (2001) 154(3):631-44. Epub 2001/08/08. doi: 10.1083/jcb.200106049. PubMed PMID: 11489920; PubMed Central PMCID: PMCPMC2196432.

20. Vieira OV, Botelho RJ, Rameh L, Brachmann SM, Matsuo T, Davidson HW, et al. Distinct roles of class I and class III phosphatidylinositol 3-kinases in phagosome formation and maturation. *J Cell Biol* (2001) 155(1):19-25. Epub 2001/10/03. doi: 10.1083/jcb.200107069. PubMed PMID: 11581283; PubMed Central PMCID: PMCPMC2150784.

21. Jeschke A, Zehethofer N, Lindner B, Krupp J, Schwudke D, Haneburger I, et al. Phosphatidylinositol 4-phosphate and phosphatidylinositol 3-phosphate regulate phagolysosome biogenesis. *Proc Natl Acad Sci U S A* (2015) 112(15):4636-41. Epub 2015/04/01. doi: 10.1073/pnas.1423456112. PubMed PMID: 25825728; PubMed Central PMCID: PMCPMC4403170.

22. Dai S, Zhang Y, Weimbs T, Yaffe MB, Zhou D. Bacteria-generated PtdIns(3)P recruits VAMP8 to facilitate phagocytosis. *Traffic* (2007) 8(10):1365-74. Epub 2007/07/25. doi: 10.1111/j.1600-0854.2007.00613.x. PubMed PMID: 17645435.

23. Bohdanowicz M, Cosio G, Backer JM, Grinstein S. Class I and class III phosphoinositide 3-kinases are required for actin polymerization that propels phagosomes. *J Cell Biol* (2010) 191(5):999-1012. Epub 2010/12/01. doi: 10.1083/jcb.201004005. PubMed PMID: 21115805; PubMed Central PMCID: PMCPMC2995177.

24. Dayam RM, Saric A, Shilliday RE, Botelho RJ. The Phosphoinositide-Gated Lysosomal Ca(2+) Channel, TRPML1, Is Required for Phagosome Maturation. *Traffic* (2015) 16(9):1010-26. Epub 2015/05/27. doi: 10.1111/tra.12303. PubMed PMID: 26010303.

25. Kim GH, Dayam RM, Prashar A, Terebiznik M, Botelho RJ. PIKfyve inhibition interferes with phagosome and endosome maturation in macrophages. *Traffic* (2014) 15(10):1143-63. Epub 2014/07/22. doi: 10.1111/tra.12199. PubMed PMID: 25041080.

26. Ragaz C, Pietsch H, Urwyler S, Tiaden A, Weber SS, Hilbi H. The Legionella pneumophila phosphatidylinositol-4 phosphate-binding type IV substrate SidC recruits endoplasmic reticulum vesicles to a replication-permissive vacuole. *Cell Microbiol* (2008) 10(12):2416-33. Epub 2008/08/05. doi: 10.1111/j.1462-5822.2008.01219.x. PubMed PMID: 18673369.

27. Sindhwani A, Arya SB, Kaur H, Jagga D, Tuli A, Sharma M. Salmonella exploits the host endolysosomal tethering factor HOPS complex to promote its intravacuolar replication. *PLoS Pathog* (2017) 13(10):e1006700. Epub 2017/10/31. doi: 10.1371/journal.ppat.1006700. PubMed PMID: 29084291; PubMed Central PMCID: PMCPMC5679646.

28. McEwan DG, Richter B, Claudi B, Wigge C, Wild P, Farhan H, et al. PLEKHM1 regulates Salmonella-containing vacuole biogenesis and infection. *Cell Host Microbe* (2015) 17(1):58-71. Epub 2014/12/17. doi: 10.1016/j.chom.2014.11.011. PubMed PMID: 25500191.

29. Guo P, Hu T, Zhang J, Jiang S, Wang X. Sequential action of Caenorhabditis elegans Rab GTPases regulates phagolysosome formation during apoptotic cell degradation. *Proc Natl Acad Sci U S A* (2010) 107(42):18016-21. Epub 2010/10/06. doi: 10.1073/pnas.1008946107. PubMed PMID: 20921409; PubMed Central PMCID: PMCPMC2964220.

30. Okai B, Lyall N, Gow NA, Bain JM, Erwig LP. Rab14 regulates maturation of macrophage phagosomes containing the fungal pathogen Candida albicans and outcome of the host-pathogen interaction. *Infect Immun* (2015) 83(4):1523-35. Epub 2015/02/04. doi: 10.1128/IAI.02917-14. PubMed PMID: 25644001; PubMed Central PMCID: PMCPMC4363425.

31. Harrison RE, Bucci C, Vieira OV, Schroer TA, Grinstein S. Phagosomes fuse with late endosomes and/or lysosomes by extension of membrane protrusions along microtubules: role of Rab7 and RILP. *Mol Cell Biol* (2003) 23(18):6494-506. Epub 2003/08/29. doi: 10.1128/mcb.23.18.6494-6506.2003. PubMed PMID: 12944476; PubMed Central PMCID: PMCPMC193691.

32. Via LE, Deretic D, Ulmer RJ, Hibler NS, Huber LA, Deretic V. Arrest of mycobacterial phagosome maturation is caused by a block in vesicle fusion between stages controlled by rab5 and rab7. *J Biol Chem* (1997) 272(20):13326-31. Epub 1997/05/16. doi: 10.1074/jbc.272.20.13326. PubMed PMID: 9148954.

33. Dingjan I, Linders PT, van den Bekerom L, Baranov MV, Halder P, Ter Beest M, et al. Oxidized phagosomal NOX2 complex is replenished from lysosomes. *J Cell Sci* (2017) 130(7):1285-98. Epub 2017/02/17. doi: 10.1242/jcs.196931. PubMed PMID: 28202687; PubMed Central PMCID: PMCPMC5399780.

34. Dingjan I, Paardekooper LM, Verboogen DRJ, von Mollard GF, Ter Beest M, van den Bogaart G. VAMP8-mediated NOX2 recruitment to endosomes is necessary for antigen release. *Eur J Cell Biol* (2017) 96(7):705-14. Epub 2017/07/10. doi: 10.1016/j.ejcb.2017.06.007. PubMed PMID: 28688576; PubMed Central PMCID: PMCPMC5641923.

35. Sakurai C, Hashimoto H, Nakanishi H, Arai S, Wada Y, Sun-Wada GH, et al. SNAP-23 regulates phagosome formation and maturation in macrophages. *Mol Biol Cell* (2012) 23(24):4849-63. Epub 2012/10/23. doi: 10.1091/mbc.E12-01-0069. PubMed PMID: 23087210; PubMed Central PMCID: PMCPMC3521691.

36. Sakurai C, Itakura M, Kinoshita D, Arai S, Hashimoto H, Wada I, et al. Phosphorylation of SNAP-23 at Ser95 causes a structural alteration and negatively regulates Fc receptor-mediated phagosome formation and maturation in macrophages. *Mol Biol Cell* (2018) 29(13):1753-62. Epub 2018/05/18. doi: 10.1091/mbc.E17-08-0523. PubMed PMID: 29771640; PubMed Central PMCID: PMCPMC6080709.

37. Becken U, Jeschke A, Veltman K, Haas A. Cell-free fusion of bacteria-containing phagosomes with endocytic compartments. *Proc Natl Acad Sci U S A* (2010) 107(48):20726-31. Epub 2010/11/13. doi: 10.1073/pnas.1007295107. PubMed PMID: 21071675; PubMed Central PMCID: PMCPMC2996438.

38. Collins RF, Schreiber AD, Grinstein S, Trimble WS. Syntaxins 13 and 7 function at distinct steps during phagocytosis. *J Immunol* (2002) 169(6):3250-6. Epub 2002/09/10. doi: 10.4049/jimmunol.169.6.3250. PubMed PMID: 12218144.

39. Matheoud D, Moradin N, Bellemare-Pelletier A, Shio MT, Hong WJ, Olivier M, et al. Leishmania evades host immunity by inhibiting antigen cross-presentation through direct cleavage of the SNARE VAMP8. *Cell Host Microbe* (2013) 14(1):15-25. Epub 2013/07/23. doi: 10.1016/j.chom.2013.06.003. PubMed PMID: 23870310.

40. Kissing S, Hermsen C, Repnik U, Nesset CK, von Bargen K, Griffiths G, et al. Vacuolar ATPase in phagosome-lysosome fusion. *J Biol Chem* (2015) 290(22):14166-80. Epub 2015/04/24. doi: 10.1074/jbc.M114.628891. PubMed PMID: 25903133; PubMed Central PMCID: PMCPMC4447986.

41. Sun-Wada GH, Tabata H, Kawamura N, Aoyama M, Wada Y. Direct recruitment of H+-ATPase from lysosomes for phagosomal acidification. *J Cell Sci* (2009) 122(Pt 14):2504-13. Epub 2009/06/25. doi: 10.1242/jcs.050443. PubMed PMID: 19549681.

42. Xia Y, Liu N, Xie X, Bi G, Ba H, Li L, et al. The macrophage-specific V-ATPase subunit ATP6V0D2 restricts inflammasome activation and bacterial infection by facilitating autophagosome-lysosome fusion. *Autophagy* (2019) 15(6):960-75. Epub 2019/01/27. doi: 10.1080/15548627.2019.1569916. PubMed PMID: 30681394; PubMed Central PMCID: PMCPMC6526827.

43. Wong D, Bach H, Sun J, Hmama Z, Av-Gay Y. Mycobacterium tuberculosis protein tyrosine phosphatase (PtpA) excludes host vacuolar-H+-ATPase to inhibit phagosome acidification. *Proc Natl Acad Sci U S A* (2011) 108(48):19371-6. Epub 2011/11/17. doi: 10.1073/pnas.1109201108. PubMed PMID: 22087003; PubMed Central PMCID: PMCPMC3228452.
